# Prognostic significance of peripheral myeloid-derived suppressor cells in advanced breast cancer

**DOI:** 10.1007/s12282-026-01831-w

**Published:** 2026-02-23

**Authors:** Haruka Kanaoka, Masayuki Nagahashi, Junko Tsuchida, Mamiko Kuroiwa, Sayaka Urano, Miki Komatsu, Shoko Yoshida, Aoi Oshiro, Gen Sugimoto, Ayumu Mitsuyoshi, Yusa Togashi, Akira Hattori, Tomoko Higuchi, Arisa Nishimukai, Masafumi Shimoda, Yasuo Miyoshi

**Affiliations:** Division of Breast and Endocrine Surgery, Department of Surgery, School of Medicine, Hyogo Medical University, 1-1 Mukogawa-cho, Nishinomiya, 663-8501 Hyogo Japan

**Keywords:** Breast neoplasm, Myeloid-derived suppressor cells, Regulatory t cells, Peripheral blood biomarker, Retrospective study

## Abstract

**Background:**

The exact roles of myeloid-derived suppressor cells (MDSCs) and regulatory T cells in the peripheral blood of individuals with advanced breast cancer undergoing chemotherapy remain unclear. This study aimed to assess peripheral blood immune-related markers, including MDSCs, in patients with advanced breast cancer treated with chemotherapy, and to examine the relationship between these immune cells and clinical outcomes. We also examined the relationship between MDSCs and peripheral blood biomarkers, including the absolute lymphocyte count and neutrophil-to-lymphocyte ratio.

**Methods:**

Seventy-three women who were treated with chemotherapy for advanced breast cancer were evaluated. Blood samples from patients at baseline and after cycle 1 treatment were subjected to flow cytometry.

**Results:**

Individuals with a lower baseline level of MDSCs demonstrated a significantly longer progression-free survival (PFS) and a tendency for enhanced overall survival (OS) compared with those with a higher level of MDSCs. The baseline level of MDSCs was a significant independent prognostic factor for PFS. Patients with a low proportion of MDSCs after cycle 1 had significantly better PFS and OS than their counterparts. The group of patients with a low proportion of MDSCs at baseline and after cycle 1 showed significantly better PFS and OS than the other groups. Patients with a low absolute lymphocyte count, an elevated neutrophil-to-lymphocyte ratio, and a reduced lymphocyte-to-monocyte ratio exhibited significantly higher MDSC levels.

**Conclusions:**

The level of MDSCs may serve as a prognostic indicator for individuals with advanced breast cancer. Peripheral blood biomarkers may reflect the proportion of circulating MDSCs.

**Supplementary Information:**

The online version contains supplementary material available at 10.1007/s12282-026-01831-w.

## Introduction

The immune system plays a crucial role in combating cancer [1, 2], impacting the effectiveness of immune checkpoint inhibitors and conventional cytotoxic chemotherapy drugs, and thus influencing the clinical outcomes for patients with breast cancer [3, 4]. For instance, tumor-infiltrating lymphocytes (lymphocytes surrounding the cancer) enhance the effectiveness of chemotherapy and are associated with patient outcomes [5–7]. Not only the local immune microenvironment of cancer but also the systemic immune environment that encompasses it influences cancer treatment, and the condition of immune cells in peripheral blood is linked to the clinical outcomes of patients undergoing chemotherapy [8]. Peripheral blood biomarkers, such as the absolute lymphocyte count (ALC) and neutrophil-to-lymphocyte ratio (NLR), are linked to the effectiveness of chemotherapy and patient prognosis [9–15]. Although these peripheral blood biomarkers are considered to reflect immune responses, the precise connection between immune cells in peripheral blood and the efficacy of chemotherapy is not yet fully understood.

In the new hallmark of cancer proposed by Hanahan, cancer can evade immune destruction by developing ways to avoid recognition and destruction by the immune system [16]. It can achieve this by hiding immune cells, suppressing immune responses, or directly interfering with immune effector cells [16]. Myeloid-derived suppressor cells (MDSCs) and regulatory T cells (Tregs) play major roles in moderating the response of the immune system [17, 18]. MDSCs are a heterogeneous population of immature myeloid-lineage cells that expand under pathological conditions and exert potent immunosuppressive effects [17]. Tregs are a type of leukocyte essential for maintaining equilibrium in the immune system and preventing the onset of autoimmune disorders [18]. Animal studies have reported that MDSCs significantly affect cancer progression by forming an immunosuppressive network, while inducing and maintaining Tregs [19, 20]. Furthermore, translational studies have started to highlight the involvement of MDSCs and Tregs in the advancement of disease among patients with breast cancer [21]. However, the exact roles of MDSCs and Tregs, along with CD8 + T cells, in the peripheral blood of patients with advanced breast cancer receiving chemotherapy are still not well understood. Additionally, the association of peripheral blood MDSCs and Tregs with common peripheral blood biomarkers that can be assessed in a standard clinical setting has rarely been investigated in patients with breast cancer and is still not well understood.

Here, we assessed MDSCs, Tregs, and CD8^+^ T cells in the peripheral blood of patients with advanced breast cancer undergoing chemotherapy, and examined the connection between these immune cells and clinical outcomes. We also examined the relationship between circulating MDSCs and Tregs and peripheral blood biomarkers.

## Patients and methods

### Study design

From May 2021 to November 2023, 90 women receiving chemotherapy for advanced or metastatic breast cancer were treated at Hyogo Medicine University Hospital. Of these, 73 participants who provided written informed consent and satisfied the following criteria were included in this retrospective study: a progression-free survival (PFS) of at least 14 days, availability of flow cytometry analysis data, and accessible blood data. The Institutional Review Board at Hyogo Medical University approved this study (approval number: 1969 and 106), and it was performed in accordance with the Declaration of Helsinki.

All participants were verified to have breast cancer through histological analysis. Samples with nuclear staining of ≥1% were classified as estrogen receptor (ER)-positive. Human epidermal growth factor receptor 2 (HER2) positivity was identified by an immunohistochemistry (IHC) score of 3 + or 2+, accompanied by positive HER2 amplification determined by in situ hybridization (ISH). HER2-low expression was identified by an IHC score of 2 + when there was no HER2 amplification as shown by ISH, or by an IHC score of 1+.

### Treatment and outcome

Each chemotherapy treatment was administered according to hospital regulations, following standard protocols. Treatment was discontinued in patients who experienced disease progression, intolerable toxicity, or death. Patients who showed disease progression, experienced intolerable side effects, or died had their treatment stopped. If patients encountered toxic side effects caused by any of these agents, the dosage was either stopped, reduced, or delayed according to a pre-established dosage adjustment plan under a physician’s guidance.

PFS was defined as the time from the initiation of chemotherapy to disease progression or death from any cause. In this retrospective study, disease progression was assessed by the treating physicians based on imaging studies performed at their discretion. Discontinuation due to adverse events without documented progression was considered censored. Overall survival (OS) referred to the timeframe from the commencement of chemotherapy to death due to any cause.

### Measurements of peripheral blood biomarkers

Counts of neutrophils, lymphocytes, monocytes, and platelets were automatically assessed using a hematology analyzer (XN-9000; Sysmex Corporation, Kobe, Japan). For each individual, the NLR was calculated by dividing the neutrophil count by the lymphocyte count. Similarly, the platelet-to-lymphocyte ratio (PLR) was obtained by dividing the platelet count by the lymphocyte count, and the lymphocyte-to-monocyte ratio (LMR) was determined by dividing the lymphocyte count by the monocyte count. The cutoff values for the ALC, NLR, PLR, and LMR were set at 1,250/µL, 3, 185.5, and 3.11, respectively [22].

### Flow cytometry analysis

We performed flow cytometry using fresh blood samples obtained from the patients. Peripheral blood was drawn into a tube, mixed with Dulbecco’s phosphate-buffered saline (D-PBS) (−) (Nacalai Tesque Inc., Kyoto, Japan), and then layered over Ficoll-Paque PLUS (Cytiva, Marlborough, MA, USA) using SepMate-50 tubes (STEMCELL Technologies, Vancouver, Canada). Then, the tubes were centrifuged at 1200×g for 20 min at 20 °C. The top layer, which contained the enriched mononuclear cells, was carefully extracted and placed into a new tube. Prior to centrifugation, phosphate-buffered saline was added to the samples for washing. Next, the tubes were spun at 300×g for 10 min at 20 °C. If the cell pellet appeared red, it was treated with 1X RBC Lysis Buffer (Thermo Fisher Scientific, Waltham, MA, USA) to remove any remaining red blood cells, followed by two washes with D-PBS (−). The cells were counted using a Countess 2 FL Automated Cell Counter (Thermo Fisher Scientific) and then used for flow cytometry analysis.

To begin, the cells were incubated with human serum AB (GemCell, Seven Hills, Australia) for 30 min at 4 °C in the dark to block. Subsequently, the cells were stained with conjugated monoclonal antibodies (mAbs) for another 30 min under the same temperature and conditions. Once staining was complete, the cells were washed with Cell Staining Buffer (BioLegend, San Diego, CA, USA) and then fixed using True-Nuclear 1x Fix Concentrate (BioLegend) for 15 min at room temperature in the dark for cell surface staining, and for 45 min at room temperature for intracellular staining. After the cell surface staining was finished, the cells intended for intracellular staining were treated with conjugated mAbs in True-Nuclear 1x Perm Buffer (BioLegend) for 30 min. Then, the stained cells were detected using a LSRFortessa X-20 cell analyzer (BD Biosciences, San Jose, CA, USA) and analyzed with FACSDiva software (BD Biosciences).

### Immune phenotypic profiles

Tregs are characterized as CD4^+^CD25^+^FoxP3^+^ cells, whereas MDSCs are identified as CD11b^+^CD14^+^CD33^+^ cells [23–25]. Flow cytometry was used to analyze immune cell subsets with the aid of specific antibodies. For the examination of T cell subsets, FITC-conjugated anti-CD4 mAb (RPA-T4, BioLegend), PE-conjugated anti-CD8α mAb (HIT8a, BioLegend), and APC-conjugated anti-CD3 mAb (HIT3a, BioLegend) were employed. In the case of Tregs, the antibodies used included Alexa Fluor 488-conjugated anti-FoxP3 mAb (clone 259D, BioLegend), PE-conjugated anti-CD25 mAb (M-A251, BioLegend), and APC-conjugated anti-CD4 mAb (RPA-T4, BioLegend). For MDSCs, the FITC-conjugated anti-CD14 mAb (63D3; BioLegend), PE-conjugated anti-CD33 mAb (WM53; BioLegend), and APC-conjugated anti-CD11b mAb (M1/70; BioLegend) were used. Isotype controls comprised Alexa Fluor 488-conjugated anti-mouse IgGk (MOPC-21, BioLegend) and PE-conjugated anti-mouse IgGk mAb (MOPC-21, BioLegend).

### Statistical analysis

CD4^+^ lymphocytes, CD8^+^ lymphocytes, MDSCs, and Tregs were categorized into low and high groups based on cutoff values derived from the receiver operating characteristic curve, calculated using the Youden index for the area under the curve (AUC). To assess PFS and OS for each group, Kaplan–Meier curves and log-rank tests were employed. A Cox proportional hazards model was used for univariable and multivariable analyses of PFS, providing hazard ratios (HRs) and 95% confidence intervals (CIs). Statistical significance was determined at *p* < 0.05 using a two-sided t-test, and all statistical analyses were performed using JMP Pro 17 software (SAS Institute Inc., Cary, NC, USA).

## Results

### Optimal cutoff values

The ideal cutoff values for PFS were 30.1 for CD4 + lymphocytes (AUC, 0.567), 4.0 for CD8 + lymphocytes (AUC, 0.512), 18.6 for MDSCs (AUC, 0.707), and 1.5 for Tregs (AUC, 0.537) (Supplementary Fig. 1).

### Clinical outcomes

**Fig. 1:**
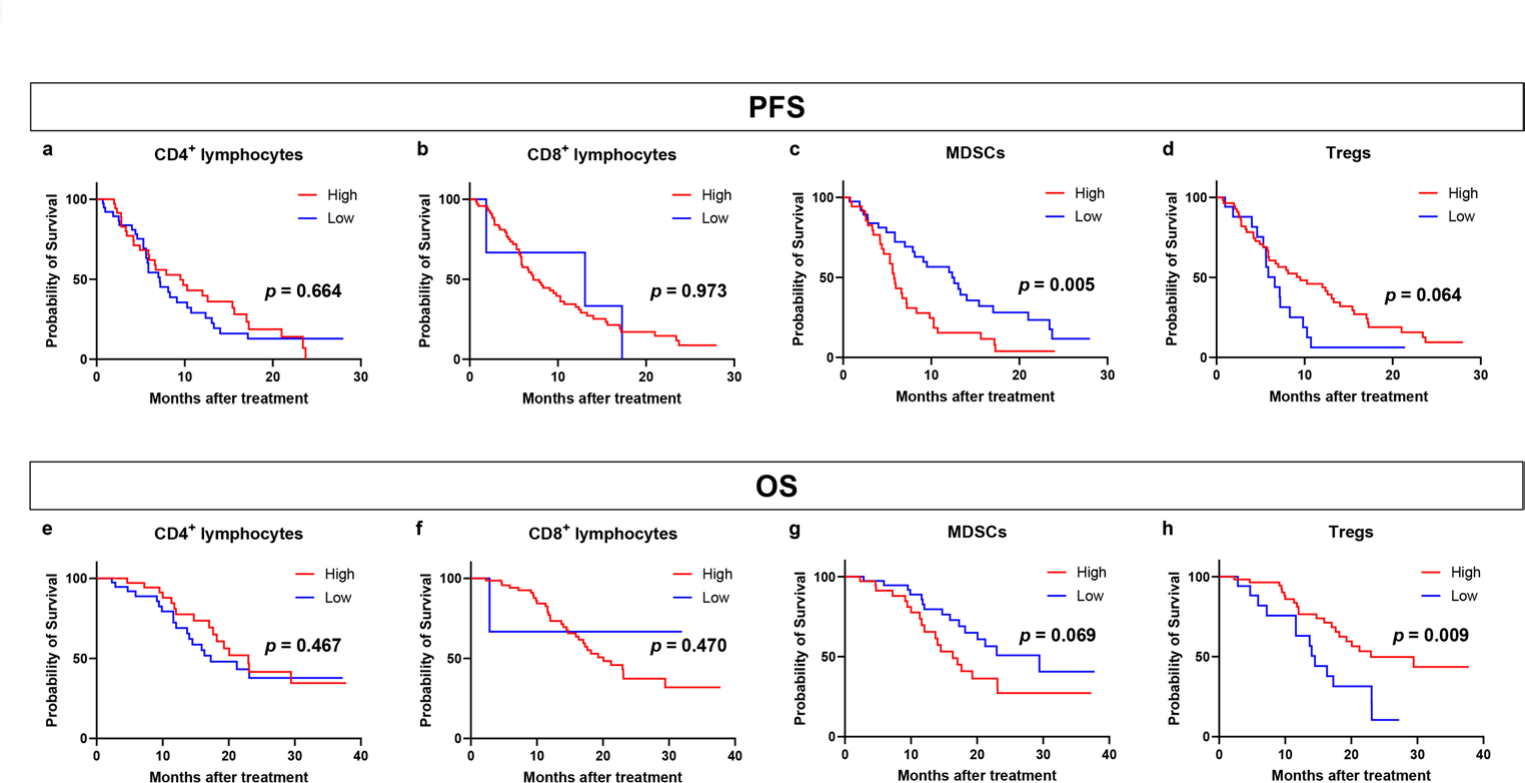
Kaplan–Meier plot of progression-free survival (PFS) in patients with breast cancer treated with chemotherapy according to the proportions of CD4^+^ and CD8^+^ lymphocytes, myeloid-derived suppressor cells (MDSCs), and regulatory T cells (Tregs) at baseline. **a**–**d** Kaplan–Meier plots of PFS in patients with breast cancer treated with chemotherapy according to the proportions of CD4^+^ lymphocytes (**a**), CD8^+^ lymphocytes (**b**), MDSCs (**c**), and Tregs (**d**). **e**–**h** Kaplan–Meier plot of overall survival (OS) in patients with breast cancer treated with chemotherapy according to the proportions of CD4^+^ lymphocytes (**e**), CD8^+^ lymphocytes (**f**), MDSCs (**g**), and Tregs (**h**)

We analyzed the clinical outcomes of 73 patients with advanced breast cancer (Supplementary Table 1). Specifically, we examined the relationship between these patients’ clinical outcomes and their baseline levels of CD4^+^ and CD8^+^ lymphocytes, MDSCs, and Tregs (Fig. 1). We first examined the relationship between immune cell levels and PFS (Fig. 1a–d). Patients with low baseline MDSC levels had significantly better PFS than those with high baseline MDSC levels (Fig. 1c; *p* = 0.005). Patients with high levels of Tregs showed a trend toward better PFS than those with low levels of Tregs (Fig. 1d; *p* = 0.064). There was no difference in PFS between patients with advanced breast cancer with high or low levels of CD4 + and CD8 + lymphocytes at baseline (Fig. 1a, b). Next, we examined the relationship between the levels of these immune cells at baseline and OS (Fig. 1e–h). Patients with high levels of Tregs at baseline had significantly better OS than those with low levels of Tregs (Fig. 1h; *p* = 0.009). Patients with low levels of MDSCs at baseline showed a trend toward better OS than those with high levels of MDSCs (Fig. 1g; *p* = 0.069). CD4 + and CD8 + lymphocytes at baseline were not associated with OS among patients with advanced breast cancer (Fig. 1e, f).

### Univariable and multivariable analyses

Given that the initial levels of MDSCs were associated with PFS in patients with advanced breast cancer receiving chemotherapy, we explored whether baseline MDSCs act as an independent prognostic marker in this group (Table 1). The univariable analysis indicated that MDSCs (high versus low) significantly predicted PFS (*p* = 0.007; Table 1). Moreover, the multivariable analysis demonstrated that baseline MDSCs were a significant independent predictor for PFS (*p* = 0.006; Table 1). Conversely, baseline Tregs did not serve as a significant independent predictor (*p* = 0.503; Table 1).

**Table 1:** Univariable and multivariable analyses results of progression-free survival

	*n*	Univariable analysis		Multivariable analysis	
		HR (95% CI)	*p*-value	HR (95% CI)	*p*-value
Menopausal status					
Premenopausal	14	1.000		1.000	
Postmenopausal	59	0.891 (0.457–1.739)	0.736	0.546 (0.249–1.199)	0.131
Primary advanced or recurrence					
Primary advanced	27	1.000		1.000	
Recurrence	46	0.781 (0.448–1.361)	0.383	0.749 (0.399–1.403)	0.366
Metastatic sites 0.877					
Non-visceral	8	1.000		1.000	
Visceral	65	1.398 (0.590–3.317)	0.447	1.082 (0.397–2.947)	0.877
Subtype					
Luminal	39	1.000		1.000	
HER2	19	0.510 (0.258–1.010)	0.053	0.698 (0.254–1.915)	0.485
Triple negative	13	0.763 (0.374–1.555)	0.456	0.682 (0.301–1.548)	0.361
Chemotherapy lines					
1	26	1.000		1.000	
≥ 2	47	1.973 (1.078–3.611)	0.028	2.060 (1.063–3.990)	0.032
Chemotherapy					
Eribulin	26	2.024 (1.088–3.765)	0.026	1.783 (0.722–4.404)	0.210
Paclitaxel+bevacizumab	16	1.665 (0.834–3.322)	0.148	1.242 (0.471–3.274)	0.662
Others	31	1.000		1.000	
MDSC level					
High	35	1.000		1.000	
Low	38	0.477 (0.280–0.813)	0.007	0.393 (0.202–0.764)	0.006
Treg level					
High	56	1.000		1.000	
Low	17	1.760 (0.959–3.229)	0.068	1.302 (0.601–2.820)	0.503

*MDSC* myeloid-derived suppressor cell, *Treg* regulatory T cell, *CI* confidence interval, *HR* hazard ratio

### PFS according to clinical factors

**Fig. 2:**
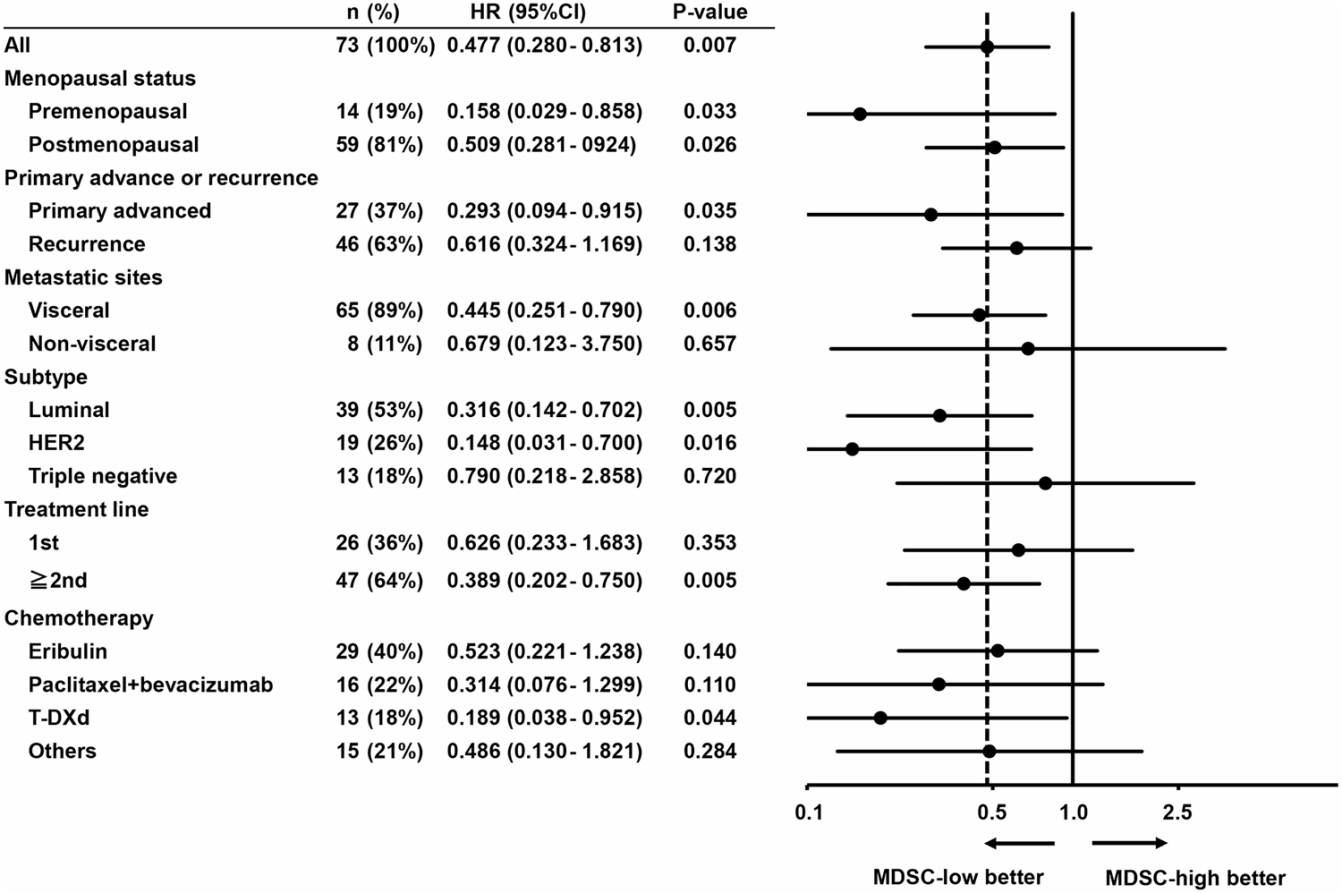
Forest plots of the proportion of myeloid-derived suppressor cells (MDSCs) at baseline for progression-free survival. The dashed line shows a hazard ratio (HR) of 0.477 in all patients. *HER2* human epidermal growth factor receptor 2, *T-DXd* trastuzumab deruxtecan, *CI* confidence interval

Given the significant correlation between the proportion of MDSCs at baseline and PFS in patients with advanced breast cancer undergoing chemotherapy, a subgroup analysis was conducted to explore this relationship (Fig. 2). Patients with a low proportion of MDSCs at baseline showed a consistently longer PFS than those with a high proportion of MDSCs (Fig. 2).

### Clinical outcomes after cycle 1 treatment

**Fig. 3:**
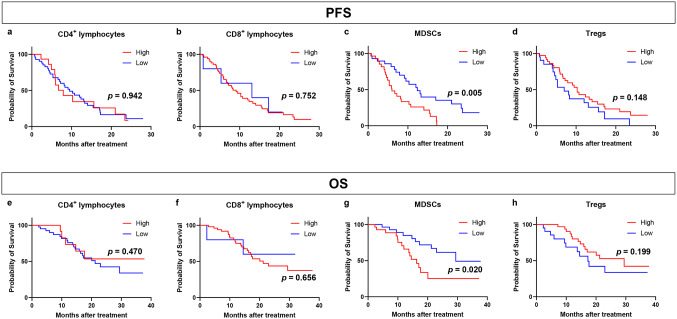
Kaplan–Meier plot of progression-free survival (PFS) in patients with breast cancer treated with chemotherapy according to the proportions of CD4^+^ and CD8^+^ lymphocytes, myeloid-derived suppressor cells (MDSCs), and regulatory T cells (Tregs) after cycle 1 treatment. **a**–**d** Kaplan–Meier plots of PFS in patients with breast cancer treated with chemotherapy according to the proportions of CD4^+^ lymphocytes (**a**), CD8^+^ lymphocytes (**b**), MDSCs (**c**), and Tregs (**d**). **e**–**h** Kaplan–Meier plot of overall survival (OS) in patients with breast cancer treated with chemotherapy according to the proportions of CD4^+^ lymphocytes (**e**), CD8^+^ lymphocytes (**f**), MDSCs (**g**), and Tregs (**h**)

Subsequently, we evaluated the link between the clinical outcomes in patients undergoing chemotherapy and the proportions of CD4^+^ and CD8^+^ lymphocytes, MDSCs, and Tregs after the first cycle of treatment (Fig. 3). We examined the relationship between the proportion of these immune cells after cycle 1 treatment and PFS (Fig. 3a–d). After the first cycle of treatment, patients with a lower percentage of MDSCs experienced significantly longer PFS than those with a higher percentage of MDSCs (Fig. 3c; *p* = 0.005). Following the initial cycle of treatment, patients with advanced breast cancer exhibited no differences in PFS, irrespective of whether their levels of CD4^+^ and CD8^+^ lymphocytes and Tregs were high or low (Fig. 3a–d). We also examined the relationship between the proportion of these immune cells after cycle 1 treatment and OS (Fig. 3e–h). Patients with a low proportion of MDSCs after cycle 1 had significantly better OS than those with a high proportion of MDSCs (Fig. 3g; *p* = 0.020). After the initial cycle of treatment, no link was found between the proportions of CD4^+^ and CD8^+^ lymphocytes and Tregs and the OS rates of patients with advanced breast cancer (Fig. 3e, f, h).

### Clinical outcomes according to changes in proportion of MDSCs

**Fig. 4:**
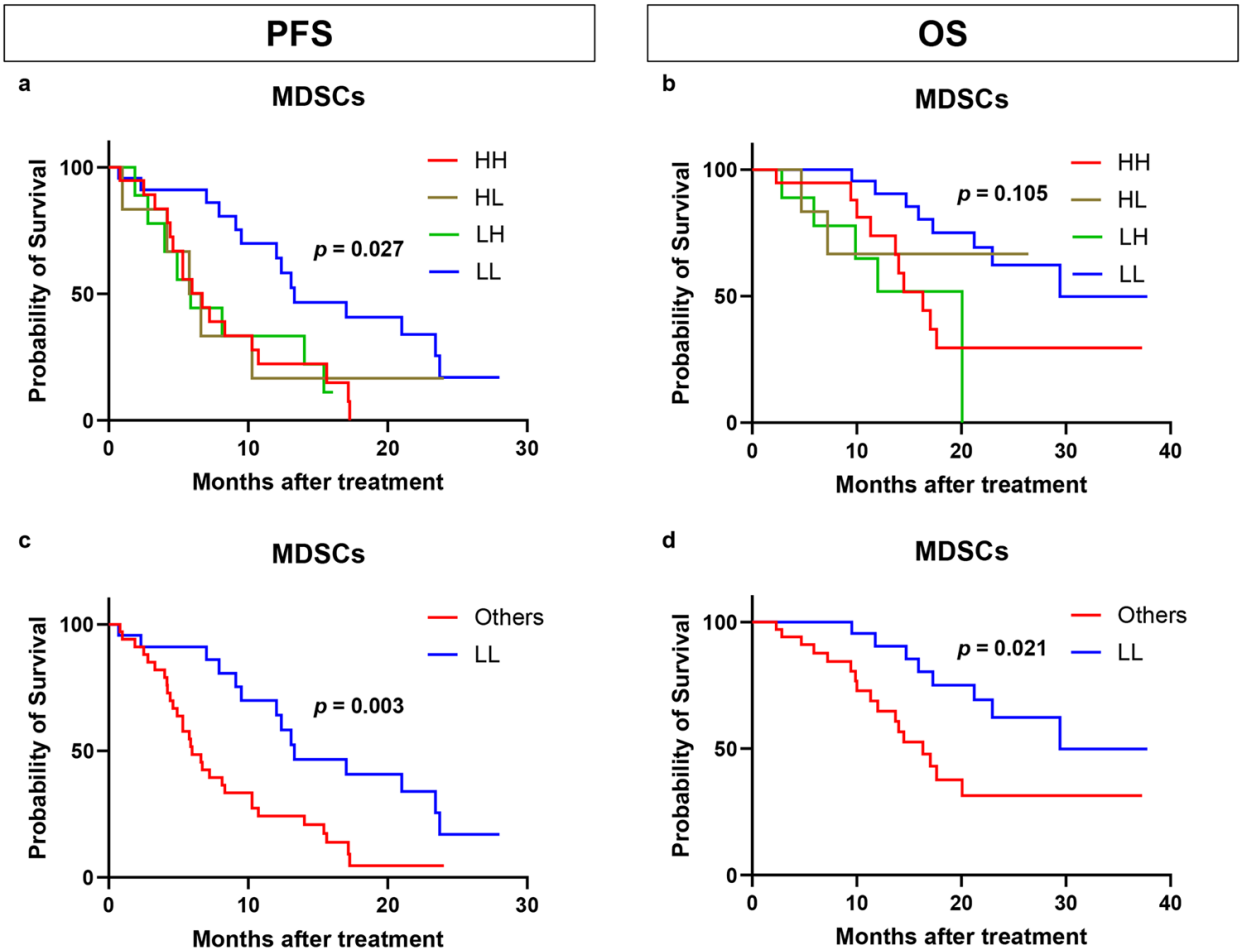
Kaplan–Meier plot of progression-free survival (PFS) and overall survival (OS) in 73 patients according to changes in myeloid-derived suppressor cell (MDSC) levels between baseline and after cycle 1 treatment. **a** Kaplan–Meier plot of PFS according to MDSCs among four groups based on changes between the baseline and cycle 1 treatment. **b** Kaplan–Meier plot of OS according to MDSCs among four groups based on changes between the baseline and cycle 1 treatment. **c** Kaplan–Meier plot of PFS according to MDSCs between the LL group and the others. **d** Kaplan–Meier plot of OS according to MDSCs between the LL group and the others. *HH* high at baseline and high after cycle 1 treatment, *HL* high at baseline and low after cycle 1 treatment, *LH* low at baseline and high at cycle 1 treatment, *LL* low at baseline and low after cycle 1 treatment

We evaluated the clinical outcomes of patients by examining the changes in the proportion of MDSCs from baseline to after the first cycle of treatment, focusing on PFS and OS (Fig. 4). Patients were divided into those with (1) a high proportion of MDSCs at baseline and post-cycle 1 (HH), (2) high proportion of MDSCs at baseline but low proportion of MDSCs after cycle 1 (HL), (3) low proportion of MDSCs at baseline and high proportion of MDSCs after cycle 1 (LH), and (4) low proportion of MDSCs at baseline and post-cycle 1 (LL). Changes in MDSCs were significantly associated with PFS (*p* = 0.027; Fig. 4a). Similarly, a trend indicating an association with OS was observed (*p* = 0.105; Fig. 4b). The LL group had the most favorable prognosis among the four groups in each analysis, prompting us to compare the prognosis of the LL group with that of the others. Consequently, the LL group demonstrated significantly longer PFS and OS than the other groups (*p* = 0.003 and *p* = 0.021, respectively; Fig. 4c, d).

### Associations of MDSCs and Tregs and peripheral blood biomarkers

**Fig. 5:**
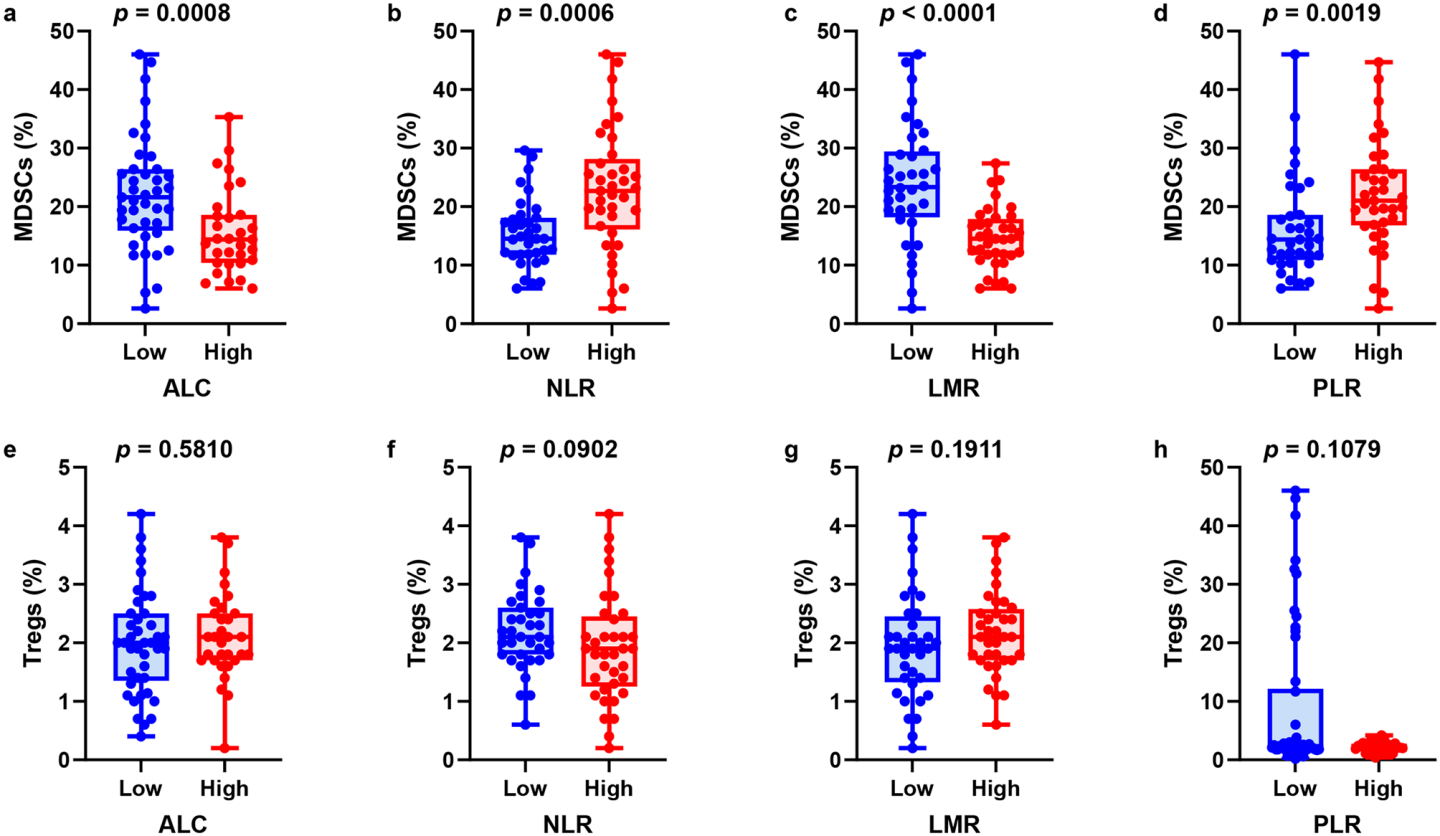
Proportions of myeloid-derived suppressor cells (MDSCs) and regulatory T cells (Tregs) according to peripheral blood biomarkers in patients with advanced breast cancer. The proportions of MDSCs (**a**–**d**) and Tregs (**e**–**h**) are shown on the basis of low and high absolute lymphocyte counts (ALCs), neutrophil-lymphocyte ratios (NLRs), lymphocyte-monocyte counts (LMRs), and platelet-lymphocyte counts (PLRs)

Because MDSCs and Tregs cannot be directly assessed in standard clinical settings, we explored the connection between peripheral blood biomarkers, which are measurable in routine tests, and MDSCs and Tregs (Fig. 5). We examined the ALC, NLR, LMR, and PLR as peripheral blood biomarkers, and the proportion of MDSCs were significantly higher in the low ALC (*p* = 0.0008; Fig. 5a), high NLR (*p* = 0.0006; Fig. 5b), low LMR (*p* < 0.0001; Fig. 5c), and high PLR groups (*p* = 0.0019; Fig. 5d). Conversely, Treg levels tended to be higher in the group with low NLR than in the group with high NLR (*p* = 0.0902; Fig. 5f); however, they showed no correlation with ALC, LMR, or PLR (Fig. 5e, f, h). No other clinical factors were associated with the MDSC proportion (Table 2).

**Table 2:** Associations between MDSCs and patient clinical characteristics

	MDSC-high	MDSC-low	*p*-value
Menopausal status			
Premenopausal	6	8	0.771
Postmenopausal	29	30	
De novo/recurrence			
De novo	16	11	0.154
Recurrence	19	27	
Metastatic site			
Visceral	30	35	0.468
Non-visceral	5	3	
Subtype			
Luminal	18	21	0.657
HER2	9	10	
Triple negative	8	5	
No. of chemotherapy lines			
1	11	15	0.625
≥2	24	23	
Chemotherapy			
Eribulin	18	11	0.216
Paclitaxel+bevacizumab	5	11	
T-DXd	6	7	
Others	6	9	

*HER2* human epidermal growth factor receptor 2, *T-DXd* trastuzumab deruxtecan, *no.* number, *MDSC* myeloid-derived suppressor cell

## Discussion

Our findings revealed that the proportion of MDSCs could serve as an independent prognostic indicator in patients undergoing chemotherapy for advanced breast cancer. Patients with a lower proportion of MDSCs at the start of and after one treatment cycle had a significantly more favorable prognosis than their counterparts. Furthermore, the levels of MDSCs were significantly associated with peripheral blood biomarkers.

In this study, we showed that MDSCs serve as an independent prognostic indicator for PFS in patients with advanced breast cancer undergoing chemotherapy. Previous studies have also shown that MDSCs act as a prognostic marker in patients with advanced breast cancer. Bailur et al.. reported that older patients with this disease who had a higher proportion of MDSCs also exhibited lower counts of circulating HER2-reactive CD8^+^ T cells, which was linked to a worse prognosis [26]. Gonda et al.. performed an analysis of MDSCs in a cohort of 155 individuals diagnosed with breast cancer, and their findings indicated that patients at stage IV who experienced an increase of more than 1% in MDSCs within their peripheral blood exhibited significantly poorer survival rates than those with a lower increase in MDSCs [27]. They proposed that the increase in MDSCs was linked to a type 2 immune response, poor nutrition, and inflammation [27]. Thus, high numbers of MDSCs are associated with a poor prognosis in patients with advanced breast cancer receiving chemotherapy.

The percentage of MDSCs following one treatment cycle and the variation in MDSCs from baseline to post-cycle were initially observed to be associated with PFS and OS in individuals with advanced breast cancer. However, further stratified analysis revealed that only patients with persistently low MDSC levels, i.e., those classified in the LL group (low MDSC levels at baseline and low MDSC levels after one cycle), demonstrated favorable survival outcomes. In contrast, patients whose MDSC levels decreased from high levels at baseline to low levels after one cycle (HL group) did not exhibit improved PFS, suggesting that a transient reduction in MDSCs may not be sufficient to confer prognostic benefit. Previous studies have reported that chemotherapy can influence circulating MDSC levels. Gunarsa et al.. reported that patients with colorectal cancer receiving oxaliplatin-containing chemotherapy who experienced early and sustained decreases in MDSC levels tended to have better prognosis than their counterparts [28]. Diaz-Montero et al.. also demonstrated that MDSC levels in peripheral blood are affected by chemotherapy and correlate with the clinical stage and tumor burden in various cancers, including breast cancer [29]. Taken together with our findings, these studies suggest that MDSC levels reflect tumor volume and host immune status, and that patients who maintain low MDSC levels both before and during treatment may have inherently better prognosis than their counterparts. Thus, persistently low MDSC levels may be more informative than treatment-induced short-term reductions in MDSC levels in predicting treatment efficacy and survival outcomes.

Furthermore, clinical studies indicated that cytotoxic chemotherapy can modulate MDSC levels; for example, anthracycline-based regimens (doxorubicin/cyclophosphamide) can induce a transient surge in circulating MDSCs [29], whereas subsequent taxane therapy may lead to a reduction of MDSC counts [30]. Certain chemotherapeutic agents like gemcitabine have even been shown to deplete MDSCs in patients with metastatic breast cancer, potentially restoring aspects of anti-tumor immunity [27]. Notably, patients with lower circulating MDSC levels (at baseline or after chemotherapy) tend to experience better treatment outcomes—for instance, higher rates of pathologic complete response and longer survival—than those with persistently high MDSC levels [27, 31].

In our study, higher baseline Treg levels were associated with better OS and a trend toward improved PFS. However, they were not independent prognostic factors, and post-cycle 1 Treg levels showed no prognostic value. This suggests that peripheral Treg levels may reflect a broader immune state rather than exerting a strong, independent effect on prognosis. Prior studies showed that higher circulating Treg levels are often associated with poor prognosis in patients with breast cancer [32, 33] and that increased Treg infiltration within tumor tissue is linked to poor outcomes in several cohorts [34–36], whereas FoxP3^+^ Treg levels are associated with improved survival in colorectal, head and neck, and esophageal cancers [35]. Furthermore, a study on *BRCA1/2*-related breast cancer reported that high levels of tumor-infiltrating CD4^+^, CD8^+^, or FOXP3^+^ cells were associated with lower mortality [37], indicating that the clinical significance of Treg levels varies by tumor subtype, anatomical compartment, and immune context. Although MDSCs promote Treg expansion and immunosuppression, in our cohort MDSCs, not Tregs, remained independently associated with poor outcomes, suggesting a more direct prognostic role. Peripheral Treg levels may instead reflect an activated or remodeled immune state, consistent with studies showing that the balance between Treg levels and CD8^+^ effector T cells is more prognostically informative than Treg levels alone [38]. Overall, while MDSCs emerged as more robust predictors of adverse outcomes, Tregs appear to play a nuanced, context-dependent role best interpreted alongside other immune subsets and features of the tumor microenvironment.

Tregs show a less consistent association with treatment response than MDSCs. The prognostic significance of Tregs varies by molecular subtype and immune microenvironment, with studies reporting both poor and variable outcomes depending on context, as aforementioned. Some evidence has suggested that intratumoral Treg abundance may be linked to chemotherapy response in specific subtypes, such as triple-negative breast cancer, but this effect is not uniformly observed across all breast cancer types [39]. Together with our findings, we conclude that Tregs have a nuanced, context-dependent relationship with chemotherapy efficacy and are more informative when assessed alongside other immune markers, such as effector T cells and MDSCs.

MDSCs were significantly associated with all peripheral blood biomarkers examined. To our knowledge, no previous study has examined the association between circulating MDSC levels and peripheral blood biomarkers that are routinely measured in clinical practice, such as ALC and NLR, in patients with breast cancer. This study is the first to demonstrate such an important association. Many studies, including ours, have reported the prognostic importance of peripheral blood biomarkers in patients with advanced breast cancer [9–15, 40, 41]. Our research findings indicate that peripheral blood biomarkers might reflect the condition of MDSCs, given the strong association observed between these biomarkers and MDSCs. Furthermore, our findings align with those of earlier research showing that alterations in peripheral blood biomarkers are linked to the prognosis of patients with breast cancer [15, 41]. The present results suggest that MDSCs are involved in at least part of the immune mechanism, as evidenced by biomarkers in the peripheral blood. Nonetheless, additional research is necessary to clarify the mechanisms that underlie the immune status reflected by the levels of these peripheral blood biomarkers.

This study has several limitations inherent to its retrospective design. First, radiological assessments and imaging intervals were not standardized and were determined by individual treating physicians, which may have introduced variability in the timing of progression evaluation. Second, patients who discontinued treatment due to adverse events were censored, which may have affected the accuracy of PFS estimation. As a result, PFS in this study may not be as strictly defined as in prospective clinical trials. These limitations should be considered when interpreting the survival analyses, and prospective studies with predefined assessment schedules are warranted to validate our findings. Additional blood samples were not successfully drawn from all eligible patients after one cycle, further reducing the proportion of cases analyzed. We analyzed MDSCs defined by the phenotype CD11b⁺CD14⁺CD33⁺ without including HLA-DR negativity or further discrimination into monocytic (CD14⁺), polymorphonuclear (CD15⁺), or early-stage (CD14⁻CD15⁻) subsets. We acknowledge that this simplified gating strategy may not fully capture the heterogeneity and functional diversity of MDSC populations. According to current consensus recommendations, accurate identification of human MDSCs requires exclusion of HLA-DR expression and further phenotypic classification into monocytic (M)-MDSC, polymorphonuclear (PMN)-MDSC, and early-stage MDSC subgroups, which exhibit distinct immunosuppressive functions and clinical relevance [42–44]. In particular, M-MDSCs are generally CD14⁺HLA-DR⁻/low, whereas PMN-MDSCs are CD15⁺CD14⁻; emerging evidence has indicated that these subsets differentially influence cancer progression and therapeutic outcomes [45–47]. However, due to limitations in sample availability and the retrospective study design, extended immunophenotyping was not feasible. Future prospective studies incorporating standardized definitions and multi-parameter flow cytometry will be necessary to validate our findings and to elucidate the distinct roles of each MDSC subset in modulating chemotherapy response. Nevertheless, this study demonstrates significant differences in clinical outcomes based on the use of MDSCs as a prognostic marker. These results are important for understanding the importance of MDSC in patients with advanced breast cancer.

In conclusion, MDSCs could be used as a prognostic factor in patients with advanced breast cancer receiving chemotherapy, as they were significantly associated with peripheral blood biomarkers.

## Supplementary Information

Below is the link to the electronic supplementary material.

Supplementary Material 1

Supplementary Material 2

## Data Availability

The datasets used in this study are available from the corresponding author upon request.
