# Supplementary material for: Prognostic significance of peripheral myeloid-derived suppressor cells in advanced breast cancer
Source: Breast Cancer. 2026 Feb 23;33(3):601–11. doi: 10.1007/s12282-026-01831-w (PMC13124773; doi:10.1007/s12282-026-01831-w)
Supplement: Supplementary file 1 — Supplementary Material 1 [file 12282_2026_1831_MOESM1_ESM.docx]

| **Supplementary Table 1**. Clinical characteristics of patients with breast cancer (*n*=73) | | |
| --- | --- | --- |
| Characteristic | *n* | (%) |
| Menopausal status |  |  |
| Premenopausal | 14 | (19.2%) |
| Postmenopausal | 59 | (80.8%) |
| De novo/recurrence |  |  |
| De novo | 27 | (37.0%) |
| Recurrence | 46 | (63.0%) |
| Metastatic site |  |  |
| Visceral | 65 | (89.0%) |
| Non-visceral | 8 | (11.0%) |
| Subtype |  |  |
| Luminal | 39 | (53.4%) |
| HER2 | 19 | (26.0%) |
| Triple negative | 13 | (17.8%) |
| Unknown | 2 | (2.7%) |
| No. of chemotherapy lines |  |  |
| 1 | 26 | (35.6%) |
| ≥2 | 47 | (64.4%) |
| Chemotherapy |  |  |
| Eribulin | 29 | (39.7%) |
| Paclitaxel+bevacizumab | 16 | (21.9%) |
| T-DXd | 13 | (17.8%) |
| Others | 15 | (20.5%) |
| HER2, human epidermal growth factor receptor 2; T-DXd, trastuzumab deruxtecan; no., number | | |
