# Supplementary figures and images for: Prognostic significance of peripheral myeloid-derived suppressor cells in advanced breast cancer
Source: Breast Cancer. 2026 Feb 23;33(3):601–11. doi: 10.1007/s12282-026-01831-w (PMC13124773; doi:10.1007/s12282-026-01831-w)

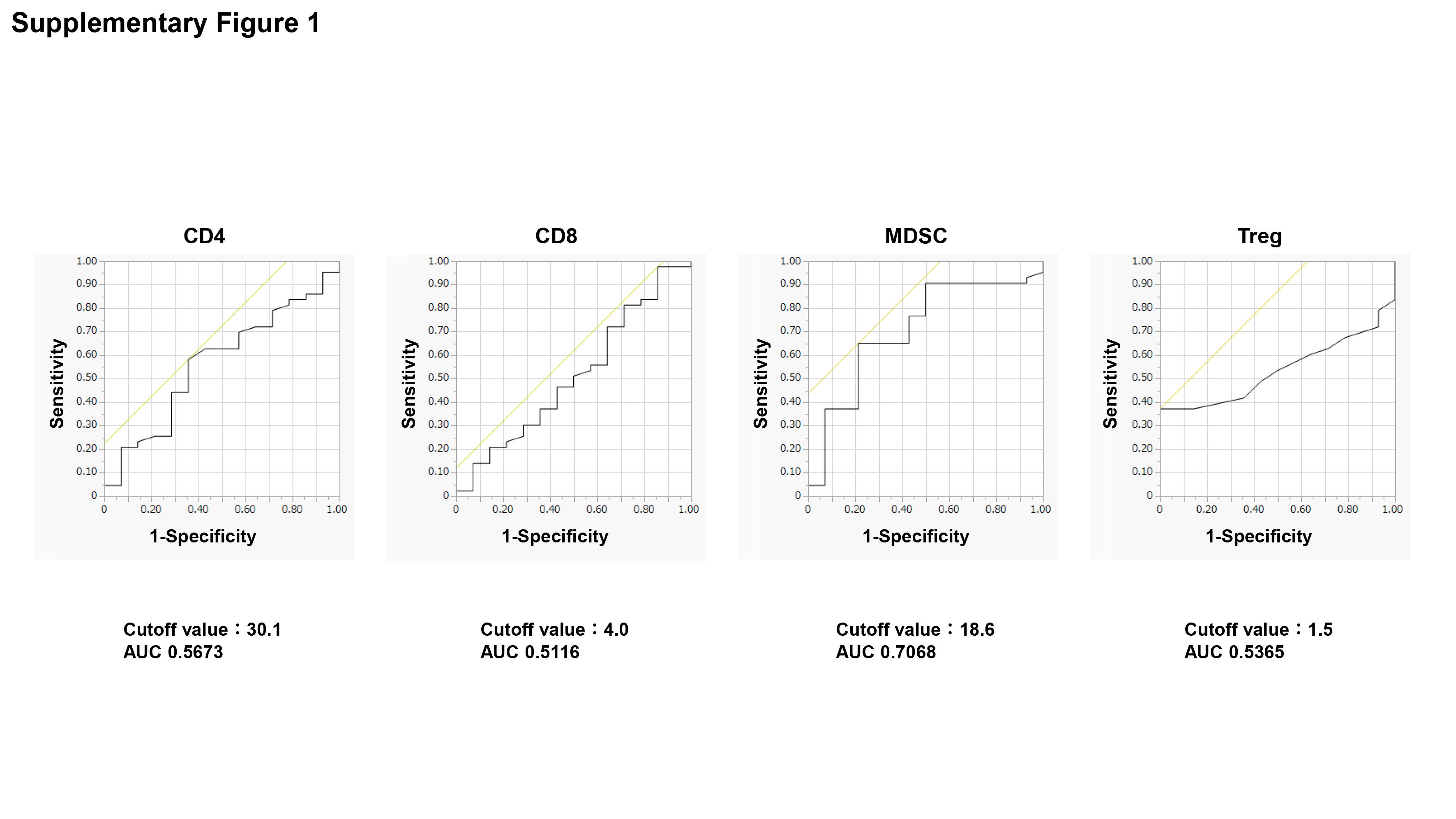

Supplement: Supplementary file 2 — Supplementary Material 2 [file 12282_2026_1831_MOESM2_ESM.tif]
